# Characteristics of Cowsheds in Vietnamese Smallholder Dairy Farms and Their Associations with Microclimate—A Preliminary Study

**DOI:** 10.3390/ani11020351

**Published:** 2021-01-30

**Authors:** Nguyen N. Bang, John B. Gaughan, Ben J. Hayes, Russell E. Lyons, Nguyen V. Chanh, Nguyen X. Trach, Duong N. Khang, David M. McNeill

**Affiliations:** 1 School of Veterinary Science, The University of Queensland, Gatton, QLD 4343, Australia; dr.russ.lyons@gmail.com; 2 Faculty of Animal Science, Vietnam National University of Agriculture, Hanoi 131000, Vietnam; nxtrach@vnua.edu.vn; 3 School of Agriculture and Food Sciences, The University of Queensland, Gatton, QLD 4343, Australia; j.gaughan@uq.edu.au; 4 Queensland Alliance for Agriculture and Food Innovation, The University of Queensland, St Lucia, QLD 4067, Australia; b.hayes@uq.edu.au; 5 Faculty of Animal Science and Veterinary Medicine, Nong Lam University, Ho Chi Minh 71308, Vietnam; chanh.nguyenvan@hcmuaf.edu.vn (N.V.C.); duongnguyenkhang@gmail.com (D.N.K.)

**Keywords:** tropical climate, heat stress, temperature humidity index, heat load, altitude, roof height

## Abstract

**Simple Summary:**

Appropriately designed cowsheds could help improve the microclimate within Vietnamese tropical smallholder dairy farms to minimise the risk of heat stress in the cows. Currently, these farmers build cowsheds on whatever land is available based on self-accumulated experiences without careful consideration of heat stress. This study characterised heat stress abatement strategies by identifying the housing parameters most associated with the cowshed microclimate across four climatically contrasting dairy regions of Vietnam. During the daytime, the microclimate inside the cowsheds was found to be relatively hot in highland and very hot in lowland regions. Although there were seven typical cowshed types defined, none were more effective than the others in improving cowshed microclimate. Increasing altitude, eave roof height and floor area per cow, and using the roof soakers together with fans, were most associated with improving microclimate, as indicated by decreasing temperature, decreasing temperature-humidity index and increasing air speed. These cowshed parameters should be prioritised for future research into the amelioration of heat stress of the cows in tropical smallholder dairy farms.

**Abstract:**

In smallholder dairy farms (SDFs), farmers often build cowsheds using local materials and based on self-accumulated experience without due consideration to reducing the risk of heat stress. This study aimed to characterise the heat stress abatement strategies and microclimate within SDF cowsheds from four typical dairy regions of Vietnam (south lowland, south highland, north lowland and north highland) and identify the housing parameters most associated with the microclimate. The study was conducted on 32 SDFs (eight SDFs per region) in autumn 2017. Twelve housing management variables, illustrating cowshed design and heat stress abatement methods of each SDF, were collected. Six microclimate parameters, collected within the cowshed, were temperature (AT), humidity, air speed (AS), heat load index (HLI), Temperature-humidity index (THI) and accumulated heat load units (AHLU) during a day (06:00 h to 18:00 h). Factor analysis and cluster analysis was applied to group cowsheds of SDFs into clusters where SDFs in the cluster had the same cowshed characteristics. Multivariable linear models were applied to define the parameters most likely to inform future research into heat stress mitigation on SDF. Averaged from 08:00 h to 18:00 h, microclimate inside the cowsheds was considered hot (HLI > 79) in the highland and very hot (HLI > 86) in the lowland regions. Cows in the lowland regions accumulated high heat load (AHLU > 50) by 18:00 h. Cowsheds of SDFs varied widely and grouped into seven cowshed types, but no type was more effective than others in reducing heat stress conditions within cowsheds. Using roof soakers together with fans decreased AT and HLI by 1.3 °C and 3.2 units, respectively, at 14:00 h compared to 11:00 h. Each 100 m increase in altitude was associated with decreases of 0.4 °C in AT, 1.3 units in HLI and 0.8 units in THI (*p* < 0.001). Each meter increase in the eave height of the cowshed roof was associated with decreases of 0.87 °C in AT, 3.31 units in HLI and 1.42 units in THI, and an increase of 0.14 m/s in AS (*p* < 0.05). The cowshed parameters that should be prioritised for future research into the amelioration of heat stress in SDF cows include using the roof soakers together with fans, increasing altitude, eave roof height and floor area per cow.

## 1. Introduction

Heat stress is an inherent difficulty associated with dairy farming in the tropics due to the hot and humid weather conditions that cows are likely to be exposed to [1,2]. Such conditions reduce cow feed intake, milk production, reproduction and negatively impacts welfare [3,4,5]. Parameters of the microclimate inside cowsheds that are often proposed as drivers of heat stress include mostly ambient temperature, humidity, air speed, solar radiation, temperature-humidity index (THI; that combines temperature and humidity) and heat load index (HLI; that combines temperature, humidity, solar radiation and air speed) [6,7,8].

In the large-scale dairies that predominate in developed countries, the risk of heat stress is managed in part by developing them in relatively cool regions (e.g., temperate or highland) using cowsheds designed to moderate THI or HLI within [9,10,11]. Compared to developing countries, developed countries have a relative abundance of land, financial resources and ease of access to cowshed design standards that optimise cow welfare [12,13,14,15,16]. Increasingly, developed countries are also required by official regulations to meet certain criteria for cow welfare [17,18,19,20,21].

In contrast, cowsheds in smallholder dairy farms (SDFs; farms with <20 lactating cows on average), which are the most common type of dairy farm in tropical Southeast Asian countries like Vietnam, vary greatly in style, size, design, construction material and equipment [22]. Cowsheds on SDFs are often built on whatever available land there is, using locally available materials rather than materials that might be more appropriate to minimise heat stress [22,23,24]. These farmers often design cowsheds based on personal experience or the accumulated experience of farmers they know, rather than official regulations designed to optimise cow welfare [24,25]. Currently, no such regulations exist in Vietnam.

Vietnam is a typical tropical country where dairy production is neither a strength nor tradition of Vietnam [26]. In the past, SDFs were mainly developed in highland regions of Vietnam to provide suitable cowshed microclimates for high yielding cows [27]. However, in recent decades there has been a shift toward the development of SDFs in lowland regions as that is where most of the human population resides and hence where the demand for fresh milk is greatest. In 2017, the total dairy herd of Vietnam was 301,649 cows [28], mainly crossbred and pure Holsteins with average daily milk yields of 14 to 15 kg/cow/day [23,29,30]. The much closer proximity to market of lowland compared to highland regions means that fresh milk can also be more cost-effectively supplied to the consumer. However, as lowland regions are likely to be much hotter than highland areas, this shift needs to be matched with further research into strategies to manage the risk of heat stress for cows in SDFs. To the best of our knowledge, no published studies specific to Vietnamese SDFs are available to guide the targeting of research interventions on optimal cowshed design for the amelioration of heat stress. Thus, the aim of this study was to (1) classify and compare housing designs relative to heat stress amelioration and the microclimate within cowsheds in typical highland compared to lowland regions of Vietnam and (2) to define the housing parameters that are most associated with improved microclimate within the cowsheds.

## 2. Materials and Methods

### 2.1. Farm Visits and Measurements of Altitude, Latitude and Microclimate Data

#### 2.1.1. Farm Visits

**Figure 1:**
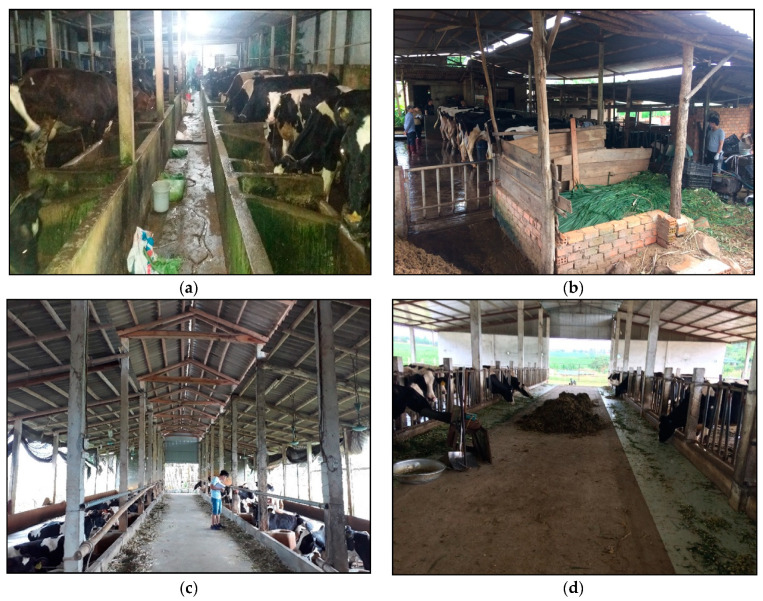
Typical interiors of smallholder dairy farms in each study region. (**a**) South lowland, (**b**) South highland, (**c**) North lowland and (**d**) North highland.

This study was conducted from 24 August to 7 October 2017 on 32 SDFs which were randomly selected from four main dairy regions (8 SDFs per region) of Vietnam including a south lowland region (SL) (10.82° N, 106.63° E), a south highland region (SH) (11.58° N, 108.14° E), a north lowland region (NL) (20.58° N and 105.92° E) and a north highland region (NH) (21.33° N, 103.91° E). These 8 SDFs per region were selected randomly from 40 SDFs per region that had previously been included in a survey of SDF economics conducted in the same year as the current study [31,32]. Each SDF was visited on an afternoon and the following morning. Examples of typical interiors of SDF in each study region are represented in Figure 1.

#### 2.1.2. Altitude, Latitude and Microclimate Data

**Figure 2:**
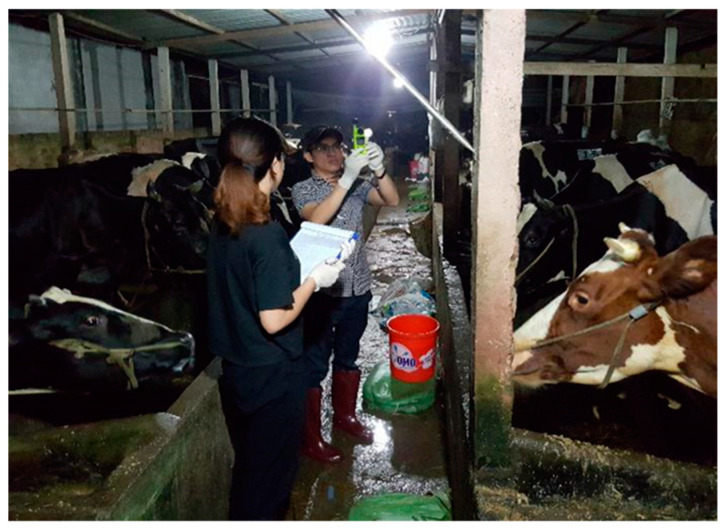
Measurement of microclimate data inside cowsheds.

The microclimate parameters inside the cowshed of each SDF were measured at 14:00 h, 16:00 h, 18:00 h (afternoon visit), 06:00 h, 08:00 h, 10:00 h and 11:00 h (morning visit) using a Kestrel 5400 Heat Stress Tracker (Nielsen-Kellerman, Boothwyn, PA, USA) in a walkway as close as possible to the middle of the cowshed, at about 1.8 m above the floor (Figure 2). The measured microclimate parameters were air speed (AS, m/s), dry-bulb temperature (AT, °C), relative humidity (RH, %), black globe temperature (GT, °C), natural aspirated wet bulb temperature (Tnawb, °C), wet bulb globe temperature (Twbg, °C), dew point temperature (Tdp, °C) and wet bulb temperature (Twb, °C). In addition, the Kestrel device was used to measure altitude (m) at each SDF. The latitudes of the SDFs were simply recorded as north or south.

Based on AT, cows were predicted to be normal when AT < 20 °C, at the heat stress threshold when 20 °C ≤ AT < 27 °C and suffering mid-severe heat stress (feed intake decreases and welfare is disturbed) when AT ≥ 27 °C [33,34,35].

Temperature-humidity index (THI; units) was calculated from AT (°C), Tdp (°C) and RH (%) using the equation of Yousef [36]:

THI = AT + (0.36 × Tdp) + 41.2, Tdp = (237.3 ° b)/(1.0 − b); b = [log(RH/100.0) + (17.27 × AT)/(237.3 + AT)]/17.27

Based on THI, cows were considered normal (i.e., no thermal stress) when THI < 68, heat stress threshold was 68 ≤ THI < 72, under mild to moderate heat stress when 72 ≤ THI < 80 or under moderate to severe heat stress when THI ≥ 80 [8].

Heat load index (HLI, units) was calculated from GT (°C), RH (%), AS (m/s) and base of the natural logarithm (*e*) using the equations of Gaughan et al. [6]:

When GT ≥ 25, HLI = 8.62 + 0.38 × RH + 1.55 × GT−0.5 × AS + *e*^(2.4−AS)^ When GT < 25, HLI = 10.66 + 2.8 × RH + 1.3 × GT−AS,

Based on HLI, the microclimate inside cowshed was categorised as cool when HLI < 70.0, moderate when 70 ≤ HLI < 77, hot when 77 ≤ HLI < 86 and very hot when HLI ≥ 86 [6].

Accumulated heat load units (AHLU; units), indicating the estimated amount of heat accumulated by the cows, were calculated using the equations of Gaughan et al. [6]. AHLU at a time point were calculated from AHLU at a previous time point, AHLU increment, and interval in hours between current and previous HLI measurements, using the equations

AHLU_Current_ = AHLU_Previous_ + AHLU_Increment_ × Interval, AHLU at 06:00 h (the first time point of measurement) = AHLU_Increment_ at 06:00 h,

If an actual calculated AHLU_Current_ was less than zero, it was set to zero, which indicates that the cow is in thermal balance.

AHLU_Increment_ were calculated from HLI at a time point, lower HLI threshold (HLI = 77) and upper HLI threshold (HLI = 86) as follows:

AHLU_Increment_ = HLI_Current_ − 77, if HLI_Current_ < 77 AHLU_Increment_ = 0, if 77 ≤ HLI_Current_ ≤ 86 AHLU_Increment_ = HLI_Current_ − 86, if HLI_Current_ > 86

Based on AHLU, the heat load that cows accumulated were categorised as low heat load when AHLU < 10, moderate heat load when 10 ≤ AHLU < 25, high heat load when 25 ≤ AHLU < 50 and very high heat load when AHLU ≥ 50 [6].

Historical climatic data were derived from the weather stations nearest to the studied regions and summarised in Table 1. Forecasted outdoor AT, RH and AS at 14:00 h, 16:00 h, 18:00 h, 06:00 h, 08:00 h, 10:00 h and 11:00 h in each region during the study period were derived from the World Weather Online Website (https://www.worldweatheronline.com/) and summarised per data collection period in Table 2. Table 1 showed that average AT and THI during the study period (August to October, autumn) were lower than those during the hottest period (May to July, summer) in all regions. Table 2 showed that during the whole study period, the day to day differences in outdoor climatic conditions in each region were not excessive. Both Table 1 and Table 2 showed that altitude (highland vs. lowland) and the latitude (north vs. south) appeared to be the main causes of differences in climatic conditions between the regions. Thus, altitude and latitude were then included in the models to evaluate the associations between housing management variables and cowshed microclimatic variables.

### 2.2. Farm Observation and Barn Measurements

The housing management dataset consisted of seven quantitative variables and five qualitative variables, which illustrated the design of the cowsheds, the facility used and the heat stress abatement methods that farmers applied for the cows. All the variables of the housing management dataset were recorded prior to afternoon milking.

The seven measured quantitative variables were (1) mat area (m^2^) per cow (abbreviated as MatCow), (2) floor area (m^2^) per cow (FloorCow), (3) roof height (m) at the highest point (RidgeHei), (4) roof height (m) at the lowest point (EaveHei), (5) percent of shed sides open (SideOpen), (6) number of fans per cow (FanCow) and (7) frequency (times) of hosing cows and floors per day (HoseCoFlo). To obtain these quantitative management data, the dimensions of the cowshed including length, width, highest point and lowest point and the dimensions of open side areas of the cowshed were measured using a rolling tape. Floor area (m^2^) per cow was calculated as the total width × length of the cowshed (including stalls, alleys and crossovers) divided by the number of cows present in the cowshed at the time of assessment. Numbers of floor mats and fans used in each SDF were counted. Almost SDFs used wall fans with fan diameters from 30 to 40 cm. The number of mats was counted and the dimensions of each mat were measured to calculate total mat area, and then divided by the number of cows to achieve mat area per cow (m^2^/cow). Percentage of cowshed sides which were open, as an indication of potential ventilation in the cowshed, was estimated by the ratio of open shed side area over the total shed side area. Frequency of washing cows and floor was obtained by both observation and asking the farmers.

The five qualitative variables recorded were (1) type of housing (Housing) was classified as tie-up housing (TieHousing) or loose housing (LooseHousing), (2) type of roof (RoofType), was classified as asbestos cement roof (AsbetosRoof) or sheet metal roof (MetalRoof), (3) roof ventilation (RoofVent) was classified as yes (YesRoofVent) if the roof had vent system or no if not (NoRoofVent), (4) cool cows by sprinklers (Sprinkler) was observed as yes (YesSprinkler) if cows were cooled by sprinklers or no if not (NoSprinkler) and (5) cool roofs by soakers (RoofCooler) was observed as yes (YesRoofCooler) if the cowshed has a soaker-cooling system above the roof or no if not (NoRoofCooler). The roof cooling system is a soaker system fitted above the roof to cool the roof when it started becoming hot, especially during noontime. Definitions of tie-up housing and loose housing were based on [39]. Tie-up housing (also called tie stalls) is the housing system where the cows are tied up by a rope, whereas loose housing is the housing system where the cows were not tied up and can move freely around group pens within the cowshed. In loose housing, the lying area for the cows can be either sharing open lounging or cubicles (also called free stalls). All qualitative management data were obtained by direct observations.

### 2.3. Data Analysis

#### 2.3.1. Statistical Comparisons

All statistics were performed using the base and additional packages of R software [40]. SDFs were the experimental unit in all analyses. Descriptive statistics for quantitative variables were calculated for each region using the ‘psych’ R package [41]. Before any statistical comparison, the normality of quantitative variables was tested using both the Shapiro–Wilk test and histograms. The results are presented as means for normally distributed quantitative variables, medians for non-normally distributed quantitative variables and frequency for categorical variables.

All variables were compared between regions. The choice of suitable tests for the comparisons of variables between regions was based on the guidelines of McDonald [42]. For variables that were found to be non-normally distributed, medians were compared by Kruskal–Wallis tests followed by Dunn post hoc tests (*p* < 0.05) using the ‘FSA’ R package [43]. For normally distributed variables, means were compared by one-way ANOVA tests followed by Tukey–Kramer tests (*p* < 0.05), using the ‘agricolae’ R package [44]. For categorical variables, frequencies of each sublevel of variables were compared by Fisher’s exact tests followed by Bonferroni-corrected pairwise Fisher’s exact tests (*p* < 0.05), using R ‘rcompanion’ package [45].

#### 2.3.2. Hierarchical Clustering on Principal Components

The hierarchical clustering on principal components (HCPC) method was applied to partition SDFs into clusters where SDFs in the same cluster had more similarity to each other in housing management than to those SDFs in other clusters [46]. Briefly, factorial analysis of mixed data method (FAMD) was applied first to transform the housing management dataset into non-correlated principal components (PCs). Then, some first PCs, which accounted for more than 70% of the total variance in the management dataset, was retained for hierarchical cluster analysis to identify an initial number of clusters [47,48]. Finally, the k-means clustering method was applied to identify an optimum number of clusters and assign SDFs into each cluster [46]. The HCPC analysis results were visualised as the dendrograms. All the multivariate statistical analyses were performed using R package ‘FactoMineR’ [49] and the results of multivariate analyses were visualised using R package ‘factoextra’ [50].

The characteristics of each management cluster were further explored by V-tests statistics [51], which compared then mean of each variable in each cluster with the mean of that variable in all clusters for quantitative variables and comparing the percentage of each category of each qualitative in each cluster to the percentage of that category in the whole the data set [48,51]. Through those comparisons, V-tests statistics could identify the advantages and disadvantages of each management cluster, thereby suggesting the management clusters with most advantages.

Although V-tests statistics could point out the management clusters with the most advanced housing management characteristics, they could not prove if the most advanced clusters were more effective than the other clusters in improving shed microclimate. Therefore, two-way ANOVA analysis was also performed to compare AT, RH, AS, THI and HLI between management clusters while accounting for the effects of altitude and latitude to assess if any management clusters were more effective than the others in improving the microclimate inside the cowsheds.

#### 2.3.3. Multivariate Linear Regression

Multivariate linear regression was performed to determine the predictor variables significantly associated with AT, RH, AS, THI and HLI inside the cowsheds. Besides housing management variables, altitude and latitude, which are the main drivers of the climatic conditions outside the cowsheds, were included as predictor variables in the models. To eliminate multicollinearity, among altitude, latitude and all housing management variables (seven quantitative and five qualitative variables), only the predictor variables with variance inflation factor (VIF) less than 5 were included in the initial multivariate models [52]. A manual backward elimination process was used to remove the variables one by one so that only the variables having *p* values of regression coefficients ≤0.1 have remained in the final models. The final models were also evaluated by examining the standardised residuals and leverage to ensure model assumptions were met [53].

## 3. Results

### 3.1. Microclimate within the Cow Sheds

Mean altitudes and microclimate parameters within the cowsheds from 06:00 h to 18:00 h in four regions are presented in Table 3. Mean altitude was highest in SH (967 m) than NH (937 m), and similarly low in SL and NL (47 m and 31 m, respectively) (*p* < 0.001). Mean RH (81.2%) and AS (0.40 m/s) were similar across regions (*p* > 0.05). The means of AT, GT, Twbg, Tdp, Twb, Tnawb, THI, HLI and AHLU in the highlands (SL and SH) were higher than those in the lowlands (SL and NL) (*p* < 0.001). However, these measurements were similar within the lowlands (SL and NL) and within the highlands (SH and NH) (*p* > 0.05).

**Figure 3:**
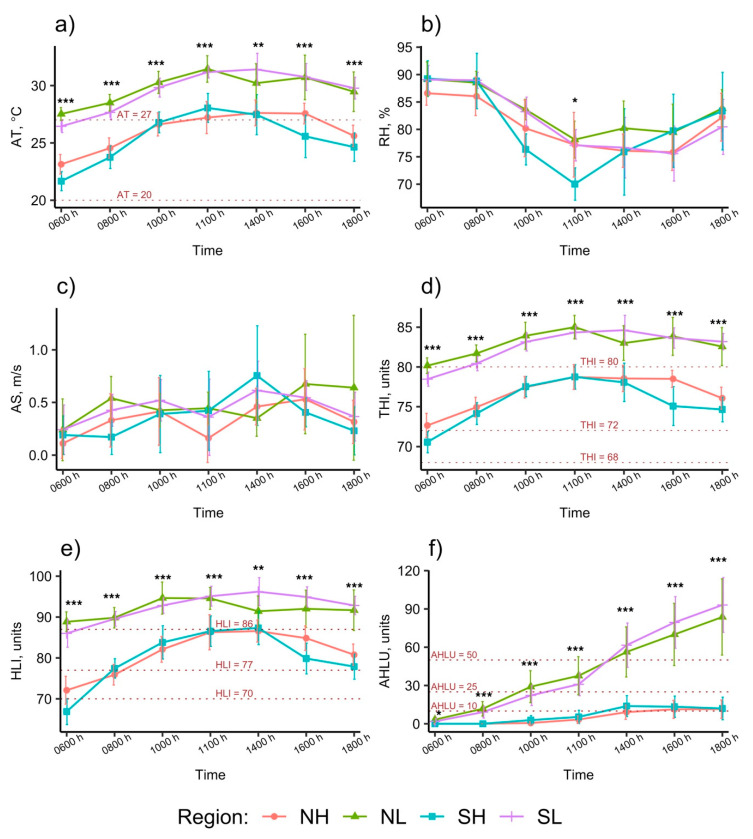
Changes of means of some main microclimate parameters in four regions during day time. (**a**) Dry-bulb temperature (AT), (**b**) Relative humidity (RH), (**c**) Air speed (AS), (**d**) Temperature-humidity index (THI), (**e**) Heat load index (HLI), (**f**) Accumulate heat load units (AHLU). Error bars represent confident intervals; Significant levels: *, *p* < 0.05; **, *p* < 0.01; ***, *p* < 0.001. AT: no thermal stress, AT < 20; heat stress threshold, 20 ≤ AT < 27; mid-severe heat stress, AT ≥ 27 [33,34,35]. THI: normal, THI < 68.0; heat stress threshold, 68 ≤ THI < 72; mild-moderate heat stress, 72 ≤ THI < 80; moderate-severe heat stress, THI ≥ 80 [8]. HLI: cool, HLI < 70; moderate, 70 ≤ HLI < 77; hot, 77 ≤ HLI < 86; very hot, HLI ≥ 86 [6]. AHLU: low, AHLU < 10; moderate, 10 ≤ AHLU < 25; high, 25 ≤ AHLU < 50; very high heat load, AHLU ≥ 50 [6].

Changes in the within-cowshed microclimate parameters during daylight hours are summarised in Figure 3. AS remained steady throughout the day and all regions showed a similar pattern. At all measurement times, mean AS were similar across regions (*p* > 0.05) (Figure 3c). The lowest mean AS was 0.12 m/s in NH SDFs at 06:00 h and the highest was 0.76 m/s in SH SDFs at 14:00 h. Across regions, mean RH was always higher than 70% during the measurement hours, highest during the period from 06:00 h to 08:00 h (87 to 89%) and lowest during the period from 11:00 h to 16:00 h (Figure 3b). Mean RH was similar across regions (*p* > 0.05) at all measurement times, except for RH at 11:00 h when mean RH in NL SDFs (78.1%) was significantly higher than that in SH SDFs (70%) (*p* = 0.034).

The interior of the cowsheds in SL and NL were classified as very hot (HLI ≥ 86) and cows were predicted to be moderately to severely heat-stressed (THI ≥ 80) throughout the day (06:00 h to 18:00 h) (Figure 3d,e). In contrast, the interiors in NH and SH were classified as hot from 08:00 h to 18:00 h (77 ≤ HLI < 86) and cows in these regions were predicted to be mild-moderate heat stress (72 ≤ THI < 80) during this period. AHLU in the highland regions increased steeply and similarly, exceeded the high heat load threshold (AHLU = 25) at approximately 10:30 h, the very high heat load threshold (AHLU = 50) at approximately 13:00 h, after which it continued to increase linearly at least until the last measurement at 18:00 h (AHLU = 83.7 units in NL and 93.1 units in SL). In contrast to the highland regions, AHLU in the lowlands increased only slightly, appearing to peak at approximately 14:00 h after which it plateaued at approximately 10 units until the last measurement (18:00 h).

Across regions, during a day the means of AT (Figure 3a), THI (Figure 3d) and HLI (Figure 3e) grouped according to highland vs. lowland, increased from 06:00 h to 11:00 h, reached the highest values from 11:00 h to 14:00 h and then started decreasing slightly. Post hoc Tukey–Kramer test showed that at 14:00 h, mean AT in NL was similar to that in NH and SH (*p* > 0.05) and mean HLI in NL was similar to that in SH (*p* > 0.05), and at 06:00 h, the mean AT in SH (21.7 °C) was lower than that in the NH (23.1 °C) (*p* < 0.001). Apart from 06:00 h and 14:00 h, at all other measurement times, means of AT, THI and HLI of SDFs in lowland regions (SL and NL) were similar (*p* > 0.05) to each other, but significantly higher (*p* < 0.05) than those measurements of the SDFs in the highlands (NH and SH).

SDFs in NL stood out from other regions when showing that during the 11:00 h to 16:00 h period, AT, HLI and THI in NL tended to decrease and reached the lowest points at 14:00 h (Figure 3a,d,e). In this region, the means of AT, HLI and THI at 14:00 h were 1.3 °C, 3.2 units and 2.5 units, respectively, lower than those at 11:00 h. The reason for the decreases of AT, HLI and THI of SDFs in NL during the hottest time of the day was the use of the soakers above the roof and fan systems. We recorded that farmers in NL turned the soakers above the roof and fan systems on at approximately 10:00 h and off at approximately 16:00 h. Farmers reported that they turned on the cooling systems for the cows when they themselves felt hot.

### 3.2. Housing Design

#### 3.2.1. Summary of Housing Management Variables

All cowsheds in NH and SH were loose housing while all in SL and 62% of cowsheds in NL were tie-up housing (*p* < 0.001) (Table 4). In all tie-up cowsheds, it was observed that cows were tied to the poles or bars adjacent to the feed and water troughs using a 1.2 to 2.0 m long rope threaded through a hole in cows’ nasal septum. Farmers reported that cows were usually tied 24 h per day for extended periods, and they were only moved to other places when they need treatments of diseases, such as lameness and metritis, or need to be moved to dry herds. Floor areas per cow were largest in NH (12.5 m^2^/cow) and similar for SH (7.5 m^2^/cow), NL (6.7 m^2^/cow) and SL (5.2 m^2^/cow) (*p* < 0.001). Use of mats (mainly polyethylene foam mats) was similar across regions (0.9 m^2^/cow, *p* = 0.698). Sheet metal roofs were most popular in SL (all eight SDFs), SH (seven out of eight SDFs), and NH (five out of eight SDFs), whereas asbestos cement roofs were most popular in NL (seven out of eight SDFs) (*p* < 0.001). Ridge roof heights were similar between regions (3.6 m, *p* = 0.118). However, eave roof height was highest in NL (3.4 m) and similar for NH (2.8 m), SL (2.6 m) and SH (2.3 m) (*p* = 0.008). All cowsheds in NL had roof vents, whereas only three in NH, one in SH, and none in SL out of 8 SDFs in each region had roof vents (<0.001).

For cooling methods, all SDFs across regions used the hose to wash the cows and floors about twice per day, usually before milking time (*p* > 0.05). While each SDF in NL had approximately eight fans for cooling the cows, each SDF in SL had only about one fan, SDFs in NH and SH did not use fans at all (*p* < 0.001). Farmers in the highlands (SH and NH) reported that they did not use any cooling methods because they thought the weather there was already very cool. Comparing between the lowland regions, SDFs in NL put more effort into cooling the cows, shown by supplying approximately one fan per cow, cooling cows by sprinklers (two out of eight SDFs) and especially cooling roof by soakers fitted above the roof (seven out of eight SDFs). In contrast, SDFs in SL used neither sprinklers to cool the cows nor soakers to cool the roof, and fans were few (1 fan for ten cows).

#### 3.2.2. Factor Analysis and Clustering Analyses

**Figure 4:**
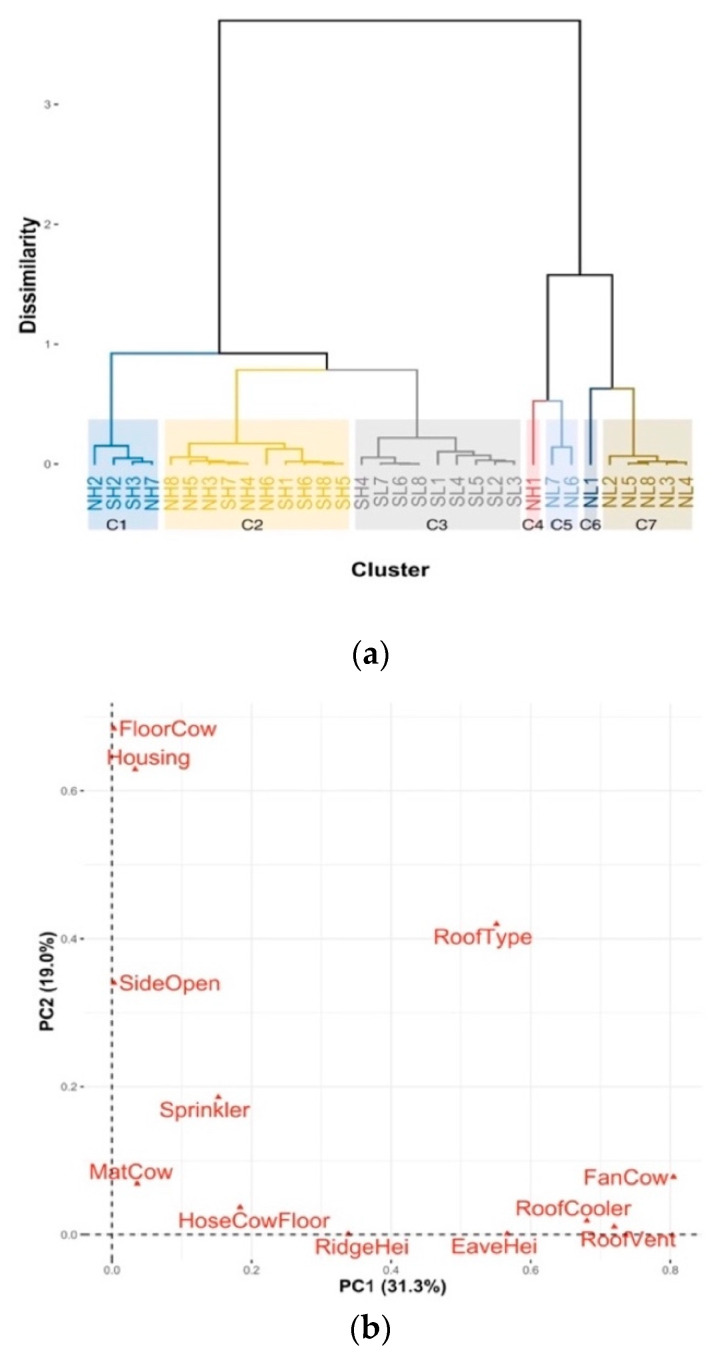

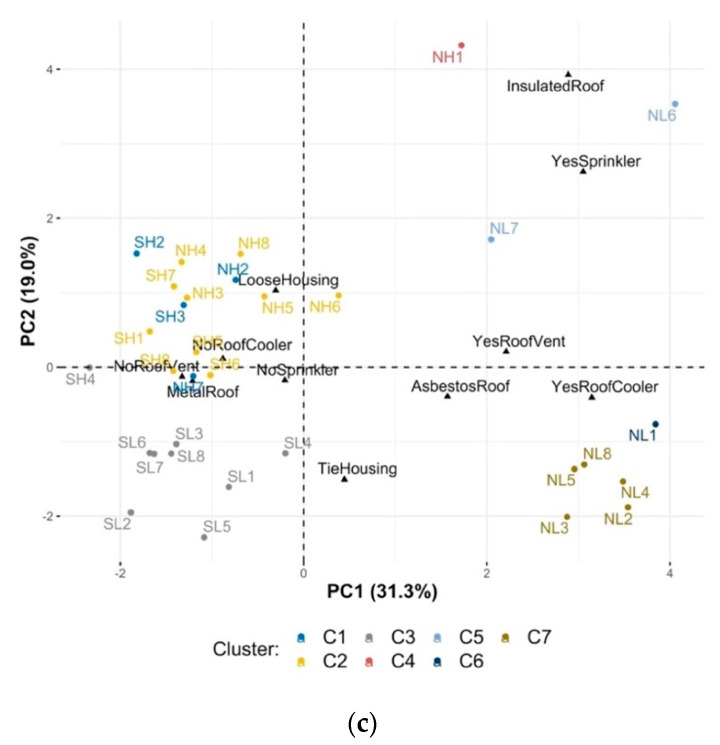
Results of factor analysis (FAMD) and hierarchical clustering on principal components (HCPC) for housing management data. Thirty-two housing conditions (or observations): SL1 to SL8, SH1 to SH8, NL1 to NL8 and NH1 to NH8 represent the housings for lactating cows in the farms numbered from 1 to 8 in south lowland, south highland, north lowland and north highland, respectively. Twelve housing management variables: MatCow, m^2^ of mat per cow; FloorCow, m^2^ of floor per cow; RidgeHei, ridge roof height (m), EaveHei, eave roof height (m); SideOpen, percent of shed sides open; FanCow, number of fans per cow; HoseCowFloor, times of hosing cow and floor per day; Housing, loose housing (LooseHousing) or tie-up housing (TieHousing); RoTyp, asbestos cement (AsbetosRoof) or sheet metal (MetalRoof); RoofVent, yes (YesRoofVent) if the roof has vent system or no if not (NoRoofVent); Sprinkler, yes (YesSprinkler) if cows are cooled by sprinklers or no if not (NoSprinkler); and RoofCooler, yes (YesRoofCooler) if the roof is cooled by soakers or no if not (NoRoofCooler). (**a**) HCPC—Cluster dendrogram. (**b**) First two PC view of all variables. (**c**) First two PC view of qualitative variables and observations.

From the housing management data on the 12 variables (Table 4), the FAMD analysis defined the first nine principal components (PCs) accounting for 79.9% of the total variance. HCPC, based on those first nine PCs, defined seven optimum housing management clusters (Figure 4a). SDFs in the same regions tended to group into the same clusters. Specifically, SDFs in SH and NH were quite similar to each other and came together into clusters C1 and C2. All SDFs in SL and one SDF in SH were in a single cluster (C3).

The directionality and amount of variation of housing management variables and the associations of these variables with the housing management clusters are presented in a 2-dimensional view of the first two principal components (Figure 4b for all variables and 4c for sublevels of qualitative variables and housing management clusters). The qualitative variables that varied the most (furthest from the original coordinates in Figure 4b,c) and most meaningful in the partitions of the clusters were “Roof type (RoTyp), asbestos cement or sheet metal”, “Cool cows with sprinklers (Sprinkler), yes or no”, “Cool roof with soakers (RoofCooler), yes or no”, “Housing, loose or tie-up” and “Cowshed has roof vents (RoofVent), yes or no”. The quantitative variables that best characterised the partition were “fans per cow (FanCow)” and “eave roof height (EaveHei)”.

The V-tests results (Table 5) showed the main characteristics of each housing management clusters by comparing the mean of each quantitative variable in each cluster with the mean of that quantitative variable in the whole dataset and comparing the percentage of categories of each qualitative variable in each cluster to the percentage of that category in the whole the dataset. Cluster 1 (two NH SDFs and two SH SDFs) had more mat area per cow, but lower “ridge roof height” than average. Cluster 2 (five SH SDFs and five NH SDFs) all had loose housing, 90% had sheet metal roof and no fans, and they had less mat area per cow but more percentage of sides open compared to average. In Cluster 3 (all eight SL SDFs and one SH SDFs), 87.5% were tie-up housing, all had sheet metal roof type, all had no roof vents and had less floor area per cow than the average. In Cluster 7 (all five were NL SDFs), all had tie-up housing, all had asbestos roof type, all had roof vents, all cooled roofs with soakers and all had higher eave roof height and more fans per cows. Figure 4a,c also show these aspects in the first two PCs.

As shown in Table 5, cowsheds in each cluster had different characteristics. When simply based on Table 5, cowsheds in Cluster 7 appear to be more advanced than the others because of having roof vents, soakers to cool the roof and more fans, and higher eave roof height. However, the results of two-way ANOVA, which compared means of AS, AT, RH, HLI or THI between housing management clusters with more than three SDFs (C1, C2, C3 and C7) while accounting for effects of the latitude and altitude, showed that none housing management clusters was more effective than the others in improving any microclimate parameter (*p* > 0.05).

### 3.3. Multivariate Models Identifying Factors Associated with Cow Shed Microclimate

The independent variables that were strongly correlated with other independent variables (VIF > 5) and therefore excluded from the initial models were: “housing, tie-up or loose”, “roof type”, “fans per cow” and “cool roofs with soakers”. The independent variables that were included in the initial models but have no significant effect were “mat area per cow”, “frequency of hosing cows and floors”, “cool cows with sprinklers” and “ridge roof height” (*p* > 0.1). A model was also fitted for RH, however, none of the variables in that model reached significance and so it is not presented.

The independent variables that were associated with AT, AS, HLI and THI are presented in Table 6. Each 100 m increase in altitude was associated with decreases of 0.4 °C in AT, 1.3 unit in HLI and 0.8 unit in THI (*p* < 0.001). Cowsheds in the south were 1.41 °C lower in AT (*p* = 0.019), 2.46 units lower in HLI (0.030) and 1.57 units lower in THI (*p* = 0.016) compared to cowsheds in the north. Each meter increase in the eave roof height was associated with decreases of 0.78 °C in AT (*p* = 0.047), 0.14 m/s in AS (*p* = 0.026), 3.31 units in HLI (*p* = 0.010) and 1.42 units in THI by (*p* = 0.011). Each m^2^ increase in floor area per cow tended to be associated with a decrease of 0.12 °C in AT (*p* = 0.094) and each 10% increase in cowshed sides open tended to be associated with a decrease of 0.5 unit in HLI (*p* = 0.052).

## 4. Discussion

As expected, cowshed microclimate was dramatically more problematic in the lowland regions. However, opportunities were also identified for improvement in the highland regions. Heat stress abatement opportunities for future research were particularly informed by some strategies employed by SDFs in the NL region.

### 4.1. Shed Microclimate

The current study, to our knowledge, was the first to directly measure HLI and THI inside cowsheds across major contrasting SDF regions in Vietnam. Based on the guidelines for HLI [6] and THI [8] to categorise level of heat stress, the very hot microclimate (HLI ≥ 86, THI ≥ 80) in the cowsheds during the daytime from 06:00 h to 18:00 h in the lowlands (SL and NL) indicated that the cows in these lowlands need to be cooled from the early morning to late afternoon of a day. Although during the day HLI and THI in the cowsheds in the highlands always maintained about 10 units lower than those in the lowlands, based on the guidelines for HLI [6] and THI [8], the cowshed microclimate in the highlands were still considered moderate hot from 07:00 h to 08:00 h (70 ≤ HLI < 77), and hot from 08:00 h to 18:00 h (77 ≤ HLI < 86, 72 ≤ THI < 80). Thus, cooling of the cows in the highlands was also necessary. Moreover, the risk of heat stress is likely to get worse at other times of the year. The current study was performed in a relatively mild time of the year, autumn, whereas microclimate can be expected to be even more extreme in the summer (Table 1) [37]. For example, Lam et al. [2], in NL SDFs, found that THI measured during early summer (May to June) averaged 81 units in the morning and 85 units in the afternoon, higher than the THI of 78.5 units at 06:00 h and 83.2 units at 18:00 h measured in the current study. These data indicate that heat stress abatement strategies need to be applied in highland as well as lowland SDFs.

The AHLU assesses heat load accumulation over time and an AHLU higher than 50 predicts that cows accumulated very high heat load [6]. In the current study, AHLU at the 18:00 h of the cowsheds in the lowlands (93.1 units in SL and 83.7 units in NL) were considerably higher than the highest threshold (AHLU = 50) suggested by Gaughan et al. [6]. These AHLUs are therefore extreme and indicate that cooling cows during day time in the lowland regions were inadequate. If cows cannot be sufficiently cooled during the daylight hours they need to be cooled at night time, to allow them to dissipate that daytime heat load to return them to their thermoneutral zone (AHLU = 0) as soon as possible [54].

The mean AS across regions (0.40 m/s) can be considered extremely low. Although not many studies have defined the optimum air speed in a cowshed, some extension websites suggested targets of between 1 to 2 m/s [55] or 2 to 3 m/s [56]. Increasing AS is important because AS was a key drivers of convection and evaporation which are the principal mechanisms for cooling cows in hot conditions [35,57]. In addition, AS is a key component in the calculation of HLI [6]. Low AS are also often associated with high AT and high RH [58,59]. Thus, the low AS could be a reason for the high RH and HLI in current study. Furthermore, the speed and pattern of airflow also directly influence air quality parameters including dust and concentrations of noxious gases such as ammonia, carbon dioxide, and methane [60]. Therefore, further research into improving air movements in cowsheds in all regions is necessary.

### 4.2. Associations between Housing Management and Cowshed Microclimate

The current study showed that although housing managements of Vietnamese SDFs varied widely to enable definition of seven clusters, the SDFs in the same region were often in the same cluster. This was expected because SDFs farmers from the same regions tend to learn cowshed design and construction from each other [24,25]. Initially, current study expected that from the diverse housing management clusters, some housing management clusters could be more effective than the others in improving microclimate. For examples, SDFs in Cluster 7 appeared better than others as the sheds in this cluster had the roof vents, soakers to cool the roof, higher eave roof height and more fans per cows. However, the results of two-way ANOVA analysis indicated that none of the housing management clusters were better than the others. This indicated that currently no SDFs had cowsheds that were optimised for improving microclimate. Thus, at the present time, the identified individual housing management variables best associated with microclimate should be relied on more than the housing management clusters to define future research directions for the abatement of heat stress in SDFs. However, in the long-term, the identification of standard housing parameters optimised for SDF cowsheds in the tropics, similar to those standards which are currently applied in commercial dairy farms [12,13,14,15,16], is necessary.

Multivariate analysis identified that altitude, latitude and eave roof height were the most important variables to focus on for the abatement of heat stress as they were all negatively associated with AT, HLI and THI. The identified decrease of 0.4 °C in AT for every 100 m increase in altitude is consistent with the finding of Trewin [61] that temperatures in the tropics typically decreases at a rate of approximately 0.6 °C per 100 m. Because AT is the main component in the calculations of HLI and THI, each 100 m increase in altitude was also associated with decreases of 1.3 unit in HLI and 0.8 unit in THI in the current study. Consequently, high altitude regions should be preferred over low altitude regions for the establishment of new SDFs where possible. However, high-altitude plateaus are few in Vietnam and virtually all of the available land in the two provinces with the largest areas of such plateaus, namely, Son La and Lam Dong, has been selected for dairy developments since late 19th century [27].

Multivariate analysis also indicated that each metre increase in eave roof height was associated with decreases of 0.78 °C in AT, 3.31 units in HLI, 1.42 units in THI and was associated with an increase of 0.14 m/s in AS. These results are consistent with those of Hatem et al. [62] who reported that increasing roof height of cowsheds from 5 to 8 m enhanced cowshed microclimate by increasing air velocities, which resulted in a decrease of maximum temperatures and an increase of milk production in Egypt. Currents results suggest that increasing roof height could be a potential intervention that would lead to a decrease in AT within the cowshed, and increase air movement through the shed. In the current study, the median eave roof heights across regions were low, ranging from 2.3 m in SH SDFs to 3.4 m in the NL SDFs. Although no studies were suggesting optimum roof heights for SDFs, the suggested roof heights for commercial large scale dairy farms are advised to be about 5 m for eave height and 9 m for ridge height, to ensure sufficient ventilation and convenience for machinery [14].

In addition to increasing eave roof height, the current study showed that the simultaneous use of roof soakers and fans were associated with reductions in AT, THI and HLI within the NL cowsheds during the hottest parts of the day (10:00 h to 16:00 h). This could be an effective strategy for heat stress abatement within SDFs in all regions, especially SL. However, those cooling systems should be turned on earlier and run for longer period.

### 4.3. Tie-Up, Floor Space and Mat Use

Optimising designs of the cowsheds is not only important in improving the microclimate, but also important in ensuring the comforts of the cows [17,18,19,20,21]. The current study identified some cowshed parameters in Vietnamese SHFs need to be improved to ensure the welfare of the cows. First, the tie-up housing occurred in all SDFs in NL and SL raised the welfare concerns because the cows would not be comfortable when they were tied by a rope threaded through a hole in cows’ nasal septum for extended periods. Compared with loose housing, tie-up housing is thought to compromise cow comfort by causing irritation and infection of the nose, causing knee and hock inflammations, reducing lying and resting time, and restricting self-grooming and social contact between cows [14,63,64]. Therefore, for Vietnamese SDFs, if possible, changing to loose housing is the best. However, if changing to loose housing is impossible, the cows should be tethered using a halter rather than a nasal rope [14].

Second, small floor area also raised welfare concerns for cows. Floor area per cow could be considered acceptable in NH (12.5 m^2^/cow), but in all other regions (5.2 to 7.5 m^2^/cow) it was too small to ensure normal cow activity. The lowest current recommended floor area per cow is 7.4 m^2^/cow, suggested in the United States in the 1980s [65]. A larger area of around 8 to 11 m^2^/cow was needed to ensure cow comfort and animal welfare standards as recommended by a number of globe welfare organisations [17,18,19,20]. In the present study, increased floor area per cows tended to be associated with decreased AT.

Third, mat use across the SDFs was also determined to be inadequate, especially in SH. The cows rested mainly on bare concrete flooring. As guidelines for cow welfare by the New Zealand Ministry of Agriculture [66] and British Columbia Society for the Prevention of Cruelty to Animals [21] highlight, bare concrete is not considered suitable. Cows should be supplied with sufficient and suitable resting surfaces, e.g., mats or bedding, or after standing on concrete surfaces for 12 h per day for three consecutive days or more, cows should be given at least one day on a comfortable surface, where they can lie down and rest freely [66]. According to this guideline, cows in NL and SL had inadequate lying conditions—that were made even worse by the cows mostly being in tied rather than loose housing.

### 4.4. Limitations

The current study had some limitations. First, the study was merely based on single day data measurements of each SDF in autumn, whereas the microclimatic conditions change seasonally or even daily. Second, the microclimate data of all 32 SDFs should have been measured on the same range of days, but current study was not able to do that due to the lack of labours and the distance between the regions. Third, while the microclimatic conditions within the cowsheds might be affected by variables such as cowshed orientation, the angle of the cowshed roofs, or roof colour [67,68]; these variables were not collected and analysed. Besides, the microclimate can vary at different positions within a given cowshed [69], thus microclimatic data should be measured at multiple points per cowshed. However, the current study only measured microclimatic data at only one point per cowshed. Further studies need to take these limitations into account to improve the accuracy of the results.

## 5. Conclusions

During the daytime, air speed inside the cowsheds across regions was very low and the microclimate inside the cowsheds across regions was hot (in highlands) to very hot (lowlands). Thus, not only SDFs in the lowlands (SL and NL), but also SDFs in the highlands (SH and NH) need to consider heat stress abatement strategies for the cows.

Although the cowsheds of SDFs varied widely and clustered into seven groups, no one group was more effective than the others in improving the microclimate inside the cowsheds. If possible, choosing the high-altitude regions to develop dairy farms, increasing the eave roof height of the cowshed height and cooling the cowshed by using the roof soakers and fans could be potential solutions to improve microclimate inside the SDF cowsheds.

To improve welfare condition for the cows, tie-up housing in SL and NL should be minimised; the floor area for cows in SL, SH and NH should be increased; and the cows across regions should be supplied with bedding materials for resting.

It is recommended that more data collection in the future study might improve the results of the study.

## Figures and Tables

**Table 1:** Average monthly rainfall, temperature (AT), humidity (RH) and temperature-humidity index (THI) during a period from 2002 to 2016 at weather stations nearest to studied regions ^A^.

Parameter	Jan	Feb	Mar	Apr	May	Jun	Jul	Aug	Sep	Oct	Nov	Dec	Average
**SL**													
Rainfall (mm)	2.3	10.8	11.1	63.5	171.9	181.7	211.3	184.0	189.3	215.4	52.9	22.5	129.6
AT, °C	25.9	26.3	27.7	29.1	29.2	28.7	28.0	28.2	27.9	27.8	27.8	26.8	27.8
RH, %	75.4	75.8	75.9	76.0	78.4	80.2	81.7	81.3	82.2	81.5	78.1	76.8	78.6
THI ^B^	75.6	76.2	78.1	80.1	80.3	79.6	78.8	79.0	78.6	78.5	78.3	76.9	78.3
**SH**													
Rainfall (mm)	8.1	24.0	77.6	167.8	225.2	201.8	225.7	244.7	306.4	235.7	93.1	33.4	156.8
AT, °C	15.9	16.9	18.1	19.2	19.7	19.4	18.9	18.8	18.7	18.3	17.9	16.7	18.2
RH, %	82.1	77.9	80.2	83.7	87.3	88.5	89.3	90.1	90.3	88.0	85.4	84.4	85.6
THI	62.4	63.5	65.3	66.8	67.6	67.3	66.7	66.6	66.4	65.8	65.2	63.5	65.6
**NL**													
Rainfall (mm)	34.9	23.5	41.2	68.1	178.6	148.6	251.5	288.0	291.8	110.6	58.8	21.5	126.4
AT, °C	16.3	18.3	20.4	24.4	27.9	29.9	29.6	28.7	27.6	25.7	22.5	18.3	24.1
RH, %	82.9	86.7	87.7	86.5	82.1	79.4	80.8	84.7	84.5	80.9	80.2	79.3	83.0
THI	62.9	65.7	68.6	74.0	78.6	81.2	80.9	79.8	78.3	75.6	71.2	65.6	73.5
**NH**													
Rainfall (mm)	37.8	20.9	46.8	120.0	170.4	200.0	276.9	264.7	146.7	53.0	37.7	34.1	119.4
AT, °C	14.7	17.6	20.5	23.7	25.1	25.7	25.3	25.1	24.3	22.3	19.1	15.7	21.6
RH, %	79.7	75.5	72.9	74.9	77.7	83.1	85.4	85.5	83.5	80.9	80.7	79.7	80.0
THI	60.7	64.4	68.3	72.6	74.7	75.6	75.2	74.9	73.8	70.9	66.7	61.9	70.0

^A^ Data were derived from General Statistics Office of Vietnam (https://www.gso.gov.vn/SLTK/) [37]; Regions: SL, South lowland; SH, South highland; NL, North lowland; NH, North highland. ^B^ THI is calculated using equations of Yousef [36].

**Table 2:** Means (SEM) of predicted outdoor temperature (AT), humidity (RH) and air speed (AS) of each region across the dates when the measurements were taken ^A^.

Region	Climatic Data Collection Period of Each Region			
	SL 24 Aug–1 Sep	SH 5–9 Sep and 3–7 Oct	NL 11–19 Sep	NH 22 Sep–1 Oct
		**AT, °C**		
SL	28.4 (0.3)	29.3 (0.4)	29.6 (0.3)	28.1 (0.3)
SH	21.0 (0.3)	21.8 (0.4)	21.9 (0.3)	20.9 (0.3)
NL	29.3 (0.3)	29.3 (0.4)	29.8 (0.3)	29.7 (0.3)
NH	27.8 (0.4)	27.5 (0.4)	28.0 (0.4)	28.6 (0.3)
		**RH, %**		
SL	78.4 (1.2)	73.6 (1.5)	72.6 (1.3)	80.5 (1.0)
SH	89.8 (1.2)	85.0 (2.0)	84.5 (1.4)	91.4 (1.1)
NL	75.8 (1.4)	76.8 (1.3)	75.0 (1.4)	73.5 (1.4)
NH	79.1 (1.6)	79.5 (1.5)	78.2 (1.6)	73.6 (1.5)
		**AS, m/s**		
SL	3.0 (0.1)	1.7 (0.1)	2.8 (0.2)	2.5 (0.2)
SH	1.1 (0.1)	0.7 (0.1)	1.2 (0.1)	0.8 (0.1)
NL	2.8 (0.2)	2.7 (0.1)	3.2 (0.3)	2.3 (0.1)
NH	1.4 (0.1)	1.3 (0.1)	1.6 (0.2)	1.5 (0.1)

^A^ Presented data were the summary of the climatic data at 14:00 h, 16:00 h, 18:00 h, 06:00 h, 08:00 h, 10:00 h and 11:00 h, which were derived from World Weather Online Website (https://www.worldweatheronline.com/) [38]; Regions: SL, South lowland; SH, South highland; NL, North lowland; NH, North highland.

**Table 3:** Comparisons of altitude and microclimate parameters (averaging from 06:00 h to 18:00 h) inside the cowsheds between four dairy regions.

Parameter ^A^	Region ^B^, Mean				*p* ^C^	Mean ± SEM
	SL	SH	NL	NH		
Altitude, m	47 ^c^	967 ^a^	31^c^	937 ^b^	<0.001	496 ± 264
AT, °C	29.5 ^a^	25.4 ^b^	29.7 ^a^	26 ^b^	<0.001	27.7 ± 1.1
RH, %	81.8	80.5	82.0	80.6	0.887	81.2 ± 0.4
AS, m/s	0.44	0.36	0.47	0.33	0.543	0.40 ± 0.03
THI, units	82.5 ^a^	75.5 ^b^	82.9 ^a^	76.7 ^b^	<0.001	79.4 ± 1.9
HLI, units	92.4 ^a^	80.0 ^b^	91.9 ^a^	81.2 ^b^	<0.001	86.4 ± 3.3
AHLU, units	42.6 ^a^	6.8 ^b^	41.7 ^a^	5.1 ^b^	<0.001	24.1 ± 10.5
GT, °C	30.0 ^a^	26.1 ^b^	29.9 ^a^	26.5 ^b^	<0.001	28.1 ± 1.0
Twbg, °C	27.5 ^a^	23.5 ^b^	27.8 ^a^	24.0 ^b^	<0.001	25.7 ± 1.1
Tdp, °C	26.4 ^a^	22.2 ^b^	26.7 ^a^	22.7 ^b^	<0.001	24.5 ± 1.2
Twb, °C	27.2 ^a^	23.1 ^b^	27.4 ^a^	23.6 ^b^	<0.001	25.3 ± 1.1
Tnawb, °C	26.6 ^a^	22.7 ^b^	27.0 ^a^	23.1 ^b^	<0.001	24.8 ± 1.1

^A^ Abbreviations: AT, dry-bulb temperature; RH, relative humidity; AS, air speed; THI, Temperature-humidity index; HLI, heat load index; AHLU, accumulate heat load units; GT globe temperature; Twbg, wet bulb globe temperature; Tdp, dew point temperature; Twb, wet bulb temperature; Tnawb, natural aspirated wet bulb temperature. ^B^ Regions: SL, South lowland; SH, South highland; NL, North lowland; NH, North highland. ^C^ *p*-values are given for One-way ANOVA tests comparing means. Superscript letters are given for post-hoc Tukey–Kramer test, *p* < 0.05. ^a–c^ Means or medians with the different superscript letters within a row differ significantly from each other, *p* < 0.05.

**Table 4:** Housing management parameters of smallholder dairy farms in four dairy regions.

Parameter	Region ^A^, Median or n ^B^				*p* ^C^	Overall ^D^
	SL	SH	NL	NH		
Qualitative variables						n (%)
Housing: Loose	0 ^b^	8 ^a^	3 ^b^	8 ^a^	<0.001	19 (59)
Housing: Tie-up	8 ^a^	0 ^b^	5 ^a^	0 ^b^		13 (41)
Roof type: Asbestos cement	0 ^b^	1 ^b^	7 ^a^	3 ^a,b^	<0.001	11 (34)
Roof: Sheet metal	8 ^a^	7 ^a^	1 ^b^	5 ^a,b^		21 (66)
Cowshed has roof vents	0 ^b^	1 ^b^	8 ^a^	3 ^a,b^	<0.001	12 (38)
Cool cows with sprinklers	0	0	2	0	0.226	2 (6)
Cool roof with soakers	0 ^b^	0 ^b^	7 ^a^	0 ^b^	<0.001	7 (22)
Quatitative variables						Mean ± SE
Floor area, m^2^/cow	5.2 ^b^	7.5 ^b^	6.7 ^b^	12.5 ^a^	<0.001	8.0 ± 1.6
Mat area, m^2^/cow	0.6	0.0	1.4	1.4	0.698	0.9 ± 0.3
Ridge roof height, m	3.3	3.3	4.1	3.6	0.118	3.6 ± 0.2
Eave roof height, m	2.6 ^b^	2.3 ^b^	3.4 ^a^	2.8 ^a,b^	0.008	2.8 ± 0.2
Shed sides open, %	75	87	75	90	0.064	81.8 ± 3.9
Fans per farm	1 ^b^	0 ^b^	8 ^a^	0 ^b^	<0.001	2.1 ± 1.8
Fans per cow	0.1 ^b^	0.0 ^b^	0.8 ^a^	0.0 ^b^	<0.001	0.2 ± 0.2
Hosing cows and floor, times/d	2	2	2	2	0.169	2 ± 0

^A^ Regions: SL, South lowland; SH, South highland; NL, North lowland; NH, North highland. ^B^ *n*, number of farms out of eight farms. ^C^ *P*-values are given for either Kruskal–Wallis tests (superscript letters are given for post hoc Wilcoxon rank sum test; *p* < 0.05) or Fisher’s exact tests (superscript letters are given for post hoc Bonferroni-corrected pairwise Fisher’s exact test; *p* < 0.05). ^D^ Overall mean (SEM) of medians or overall frequency (percentage) of all farms. ^a,b,c^ Medians or percentages with the different superscript letters within a row differ significantly from each other, *p* < 0.05.

**Table 5:** Most significant variables characterising each housing management clusters.

Cluster	Most Significant Variables	Group Mean (SD) or %	Overall Mean (SD) or %	V-Test	*p* ^A^
C1	Mat area, m^2^/cow	2.88 (0.82)	1.17 (1.09)	3.75	<0.001
	Ridge roof height, m	3.00 (0.25)	3.71 (0.74)	−2.31	0.021
C2	Shed sides open, %	88.25 (8.54)	74.92 (20.12)	2.49	0.013
	Mat area, m^2^/cow	0.57 (0.76)	1.17 (1.09)	−2.08	0.038
	Fans per cow	0.00 (0.00)	0.23 (0.34)	−2.47	0.014
	Housing = Loose	100	59.38	3.19	0.001
	Roof type = Sheet metal	90.00	59.38	2.3	0.021
C3	Floor area, m^2^/cow	5.31 (1.01)	8.67 (4.24)	−2.55	0.011
	Housing = Tie-up	87.50	40.63	2.93	0.003
	Roof type = Sheet metal	100	59.38	2.69	0.007
	Cowshed has roof vents =No	100	62.5	2.51	0.012
	Housing = Loose	12.5	59.38	−2.93	0.003
C4	Floor area, m^2^/cow	21.26 (0.00)	8.67 (4.24)	2.97	0.003
C5	Cool cows with sprinklers = Yes	100	6.25	3.09	0.002
	Cool roof with soakers = Yes	100	21.88	2.03	0.042
C6	Hosing cows and floor	5.00 (0.00)	2.13 (0.6)	4.8	<0.001
	Ridge roof height, m	6.00 (0.00)	3.71 (0.74)	3.09	0.002
	Fans per cow	1.00 (0.00)	0.23 (0.34)	2.25	0.024
C7	Fans per cow	0.79 (0.16)	0.23 (0.34)	3.96	<0.001
	Eave roof height, m	3.59 (0.31)	2.84 (0.61)	2.94	0.003
	Cool roof with soakers = Yes	100	21.88	3.88	<0.001
	Roof type = Asbestos cement	100	34.38	3.05	0.002
	Cowshed has roof vents =Yes	100	37.5	2.88	0.004
	Housing = Tie-up	100	40.63	2.73	0.006

^A^*p* values were from V-tests which compared the mean of each quantitative variable in each cluster with the mean of that variable in the whole the dataset or compared the percentage of each category of each qualitative in each cluster with percentage of that category in the whole the dataset [48].

**Table 6:** Multivariate models identifying the factors associated with the temperature (AT, °C), humidity (RH, %), air speed (AS, m/s), heat load index (HLI) and temperature-humidity index (THI) inside the cowsheds ^A^.

Variable	AT		AS		HLI		THI	
	Coef (SE) ^B^	*p* ^C^	Coef (SE)	*p*	Coef (SE)	*p*	Coef (SE)	*p*
Intercept	33.86 (1.56)	<0.001	0.02 (0.17)	0.916	107.1 (3.41)	<0.001	88.01 (1.81)	<0.001
Altitude, m	−0.004 (0.001)	<0.001	--	ns	−0.013 (0.001)	<0.001	−0.008 (0.001)	<0.001
Latitude: North	Reference				Reference		Reference	
Latitude: South	−1.43 (0.58)	0.019	--	ns	−2.46 (1.08)	0.030	−1.57 (0.61)	0.016
Eave roof height, m	−0.87 (0.41)	0.047	0.14 (0.06)	0.026	−3.31 (0.93)	0.001	−1.42 (0.52)	0.011
Floor area, m^2^/cow	−0.12 (0.07)	0.094	--	ns	--	ns	--	ns
Shed sides open, %	--	ns	--	ns	−0.05 (0.02)	0.052	--	ns
R^2^, %	79		15		88		86	

^A^ A model was also fitted for cowshed humidity (RH), however, none variable showed a significant association; In all models, the independent variables that were excluded due to VIF > 5 were: “housing”, “roof type”, “fans per cow” and “cool roof soakers”. ^B^ Coef (SE), Coefficient (Standard error). ^C^ The independent variables that were included in each model but have no significant effect (*p* > 0.1) were: “mat area”, “frequency of hosing cows and floors”, “cool cows with sprinklers”, “roof vents”, “ridge roof height” and the variables with ‘ns’ sign in *p* column of each model.

## Data Availability

The data presented in this study are available on request from the corresponding author.
